# Mitochondrial Dysfunction in Chronic Obstructive Pulmonary Disease: Unraveling the Molecular Nexus

**DOI:** 10.3390/biomedicines12040814

**Published:** 2024-04-07

**Authors:** Chin-Ling Li, Jui-Fang Liu, Shih-Feng Liu

**Affiliations:** 1 Department of Respiratory Therapy, Kaohsiung Chang Gung Memorial Hospital, Kaohsiung 833, Taiwan; 2 Department of Respiratory Care, Chang Gung University of Science and Technology, Chiayi 600, Taiwan; 3 Chronic Diseases and Health Promotion Research Center, Chang Gung University of Science and Technology, Chiayi 600, Taiwan; 4 Division of Pulmonary and Critical Care Medicine, Department of Internal Medicine, Kaohsiung Chang Gung Memorial Hospital, Kaohsiung 833, Taiwan; 5 College of Medicine, Chang Gung University, Taoyuan 333, Taiwan

**Keywords:** COPD, mitochondrial dysfunction

## Abstract

Chronic obstructive pulmonary disease (COPD) is a prevalent and debilitating respiratory disorder characterized by persistent airflow limitation and chronic inflammation. In recent years, the role of mitochondrial dysfunction in COPD pathogenesis has emerged as a focal point of investigation. This review endeavors to unravel the molecular nexus between mitochondrial dysfunction and COPD, delving into the intricate interplay of oxidative stress, bioenergetic impairment, mitochondrial genetics, and downstream cellular consequences. Oxidative stress, a consequence of mitochondrial dysfunction, is explored as a driving force behind inflammation, exacerbating the intricate cascade of events leading to COPD progression. Bioenergetic impairment sheds light on the systemic consequences of mitochondrial dysfunction, impacting cellular functions and contributing to the overall energy imbalance observed in COPD patients. This review navigates through the genetic landscape, elucidating the role of mitochondrial DNA mutations, variations, and haplogroups in COPD susceptibility and severity. Cellular consequences, including apoptosis, autophagy, and cellular senescence, are examined, providing insights into the intricate mechanisms by which mitochondrial dysfunction influences COPD pathology. Therapeutic implications, spanning antioxidant strategies, mitochondria-targeted compounds, and lifestyle modifications, are discussed in the context of translational research. Important future directions include identifying novel biomarkers, advancing mitochondria-targeted therapies, and embracing patient-centric approaches to redefine COPD management. This abstract provides a comprehensive overview of our review, offering a roadmap for understanding and addressing the molecular nexus between mitochondrial dysfunction and COPD, with potential implications for precision medicine and improved patient outcomes.

## 1. Introduction

Chronic obstructive pulmonary disease (COPD) represents a formidable global health challenge, imposing a substantial burden on individuals and healthcare systems worldwide. This progressive respiratory disorder is characterized by persistent and often irreversible airflow limitation, accompanied by chronic inflammation of the airways and lung parenchyma [1]. Despite significant strides in understanding COPD’s etiology and management, the multifaceted nature of this disease necessitates ongoing research to uncover novel insights and therapeutic targets. While traditional risk factors for COPD, such as cigarette smoking and environmental exposure, have long been recognized [1,2], recent investigations have expanded our understanding of COPD pathogenesis by shedding light on the intricate involvement of mitochondrial dysfunction [3]. Mitochondria, the cellular powerhouses responsible for energy production, have emerged as central players in the complex interplay of molecular events that drive COPD progression.

Mitochondrial dysfunction, encompassing aberrations in the structure and function of these organelles, profoundly impacts key cellular processes. The relevance of mitochondria in COPD is underscored by their involvement in oxidative stress, a hallmark feature of the disease [4]. Reactive oxygen species (ROS), byproducts of mitochondrial respiration, not only contribute to oxidative stress but also set in motion a cascade of events that perpetuate inflammation, cellular damage, and tissue remodeling [5,6].

This manuscript seeks to present a comprehensive exploration of the intricate relationship between mitochondrial dysfunction and COPD. By delving into the molecular mechanisms underlying this association, we aim to elucidate how mitochondrial abnormalities contribute to the initiation, progression, and exacerbation of COPD. Understanding these mechanisms is paramount to identifying potential therapeutic targets and developing strategies to mitigate disease burden [7].

As we embark on this journey through the molecular landscape of COPD and mitochondria, it becomes evident that unraveling these complexities holds promise for advancing our knowledge of COPD pathophysiology. By shedding light on the role of mitochondrial dysfunction in COPD, we aspire to pave the way for innovative interventions, personalized treatment approaches, and ultimately, improved outcomes for individuals grappling with this debilitating respiratory condition. The integration of mitochondrial insights into the broader context of COPD research represents a crucial step toward a more nuanced understanding of the disease and a pathway to transformative advancements in its management.

## 2. Mitochondrial Dysfunction in COPD

Mitochondrial dysfunction stands as a pivotal player in the intricate molecular landscape, contributing to the pathogenesis of chronic obstructive pulmonary disease (COPD). This section aims to provide a comprehensive exploration of the multifaceted aspects of mitochondrial involvement in COPD, with a particular emphasis on its impact on oxidative stress and bioenergetic impairment.

### 2.1. Oxidative Stress

The mitochondrion, traditionally celebrated as the cell’s powerhouse, assumes a paradoxical role in COPD by being both a source and victim of oxidative stress. Within mitochondria, the electron transport chain serves as a primary site for the generation of reactive oxygen species (ROS). In COPD, the delicate balance between ROS production and the antioxidant defense system is disrupted, culminating in an overwhelming burden of oxidative stress. Studies have unequivocally demonstrated an upsurge in mitochondrial ROS production in COPD patients, notably in response to environmental insults such as cigarette smoke [8]. Elevated ROS levels trigger a cascade of events, including the activation of inflammatory pathways and the perpetuation of oxidative damage to cellular components [9,10]. Moreover, mitochondrial DNA (mtDNA) has been found to be highly susceptible to oxidative stress, resulting in mutations and impairments in respiratory chain function. This sets the stage for a vicious cycle of mitochondrial dysfunction and increased ROS generation [11,12]. Understanding the intricate interplay between mitochondrial dysfunction and oxidative stress becomes pivotal in unraveling the mysteries of COPD pathology. Therapeutic strategies aimed at targeting mitochondrial ROS production or enhancing antioxidant defenses hold promise as novel approaches to mitigating COPD progression [13,14]. These interventions, if successful, could potentially disrupt the vicious cycle of oxidative stress and contribute to the development of more effective therapeutic modalities for COPD patients. As research advances, uncovering the precise molecular mechanisms underlying mitochondrial dysfunction in oxidative stress may pave the way for targeted and personalized treatment strategies, ushering in a new era of COPD management.

### 2.2. Bioenergetic Impairment

The disruption of the energetic equilibrium maintained by mitochondria emerges as a crucial factor in the pathophysiology of COPD, contributing significantly to the observed cellular dysfunction [15,16]. Bioenergetic impairment in COPD involves alterations in mitochondrial respiration and adenosine triphosphate (ATP) synthesis, identified as key components of this dysregulation [15,16,17,18,19]. Mitochondrial respiratory chain dysfunction, often characterized by reduced activity in electron transport chain complexes, results in an inadequate supply of ATP. This bioenergetic deficit has profound implications for cellular functions, extending to impaired mucociliary clearance, compromised immune responses, and a diminished capacity for tissue repair. Furthermore, skeletal muscle dysfunction, a prevalent comorbidity in COPD, is influenced by bioenergetic impairment, contributing to exercise intolerance and systemic manifestations of the disease [17]. Exploration into the bioenergetic aspects of mitochondrial dysfunction in COPD has revealed potential therapeutic targets. Strategies aimed at restoring mitochondrial function, such as the use of mitochondria-targeted antioxidants or agents promoting mitochondrial biogenesis, hold promise for ameliorating the bioenergetic deficits associated with COPD.

### 2.3. Integrative Approaches

The orchestration of mitochondrial dysfunction with other cellular processes, notably inflammation and cellular senescence, underscores its pivotal role in COPD pathogenesis. Mitochondrial dysfunction contributes to an aberrant inflammatory response, perpetuating a pro-inflammatory milieu in the airways and lung parenchyma. Additionally, the induction of cellular senescence, a state of irreversible growth arrest, is intricately linked to mitochondrial dysfunction and is observed in COPD. Understanding the integrative aspects of mitochondrial dysfunction in COPD provides a more comprehensive view of disease mechanisms. Targeting multiple facets of this dysfunction, including oxidative stress, bioenergetic impairment, and associated inflammatory responses, may yield more effective therapeutic interventions [20]. The exploration of integrative approaches represents a promising avenue for developing novel treatments that address the complex interplay of molecular pathways involved in COPD progression. Future therapeutic strategies may benefit from simultaneously targeting multiple aspects of mitochondrial dysfunction to achieve synergistic effects and enhance overall treatment efficacy.

## 3. Mitochondrial Genetics and Potential Mechanisms in COPD Development

The role of mitochondrial genetics in the intricate tapestry of COPD expands our understanding beyond conventional genetic factors associated with nuclear DNA. Mitochondrial DNA (mtDNA), a small circular genome located within the mitochondria, emerges as a critical player in COPD susceptibility and severity.

### 3.1. MtDNA Mutations and Variations and Their Role in COPD

Numerous studies have unveiled a compelling association between mtDNA mutations and the risk of developing COPD [21,22]. Identifying specific mtDNA mutations linked to COPD susceptibility reveals a promising avenue for comprehending individualized risk profiles and disease progression. Beyond mutations, variations in mtDNA, including single-nucleotide polymorphisms (SNPs), and mitochondrial haplogroups have also been implicated in COPD pathogenesis. These variations can influence mitochondrial function, affecting respiratory chain efficiency and overall cellular metabolism. Investigations into the relationship between mtDNA variations, haplogroups, and COPD offer valuable insights into the heterogeneity observed in disease presentation and progression [23,24]. Zheng et al. demonstrated a role of mitochondrial DNA (mtDNA) haplogroups in chronic obstructive pulmonary disease (COPD) susceptibility in a Han Chinese population, revealing that haplogroups A and M7 may increase the risk of COPD, while haplogroups D, F, and M9 might decrease the risk [24,25]. Additionally, the presence of an 822 bp mtDNA deletion in male cigarette-smoking subjects suggested that haplogroup D could heighten susceptibility to DNA damage from external reactive oxygen species, highlighting the interplay between genetic and environmental factors in COPD development. Liu et al. demonstrated that COPD patients have a lower leukocyte mtDNA copy number than non-smoker or non-COPD patients [26]. His study also showed that the IL-13 promoter (−1055) polymorphism is associated with leukocyte mtDNA copy number [27]. The sequencing of a complete mitochondrial genome of a rat chronic obstructive pulmonary disease strain, with a length of 16,310 bp and coding for 13 protein-coding genes, two ribosomal RNA genes, and 22 transfer RNA genes, provides valuable insights into potential mitochondrial DNA mutations as markers in cancer and other diseases [28]. Lin et al.’s study elucidates aberrant long non-coding RNA (lncRNA) profiles in the lung tissue of rats with chronic obstructive pulmonary disease (COPD) induced by cigarette smoke (CS) or fine particulate matter (PM2.5), revealing specific lncRNAs associated with mitochondrial dysfunction and inflammatory responses [29]. The cumulative impact of these mutations may impair mitochondrial function, leading to heightened oxidative stress and energy imbalance—both hallmark features of COPD [21,22].

### 3.2. Implications for Precision Medicine

The integration of mitochondrial genetics into COPD studies represents a paradigm shift toward precision medicine, acknowledging the role of an individual’s genetic landscape in disease manifestation. Incorporating mtDNA analyses into risk assessment models may enhance predictive accuracy, enabling the identification of individuals predisposed to COPD development or rapid progression. Moreover, mitochondrial genetics may influence treatment responses. Variations in mtDNA could impact the efficacy of interventions targeting mitochondrial dysfunction, such as antioxidant therapies or agents promoting mitochondrial biogenesis. Tailoring treatment approaches based on an individual’s mitochondrial genetic profile holds promise for optimizing therapeutic outcomes and improving patient care in COPD [25].

### 3.3. Challenges and Future Directions

Despite considerable advancements, challenges in unraveling the complex interplay between mitochondrial genetics and COPD persist. Large-scale, multicenter studies are essential to validate findings and establish robust associations. Additionally, understanding the interactions between nuclear and mitochondrial genomes and their collective impact on COPD susceptibility requires further exploration [27]. As we delve deeper into the realm of mitochondrial genetics in COPD, integrating these insights with clinical parameters and environmental influences becomes crucial. The evolving landscape of precision medicine holds the potential to revolutionize COPD management, offering tailored strategies that account for the unique genetic signatures contributing to this heterogeneous respiratory disorder. Future research endeavors should prioritize addressing these challenges, paving the way for more targeted and effective interventions in COPD management.

## 4. Cellular Consequences of Mitochondrial Dysfunction in COPD

Mitochondrial dysfunction in chronic obstructive pulmonary disease (COPD) reverberates through intricate cellular processes, influencing apoptosis, autophagy, and cellular senescence. Understanding these cellular consequences provides a nuanced perspective on how mitochondrial abnormalities contribute to COPD pathogenesis.

### 4.1. Apoptosis

Mitochondria, as central players in the regulation of apoptosis, govern a programmed cell death mechanism crucial for tissue homeostasis. In COPD, mitochondrial dysfunction disrupts the delicate balance between pro-apoptotic and anti-apoptotic signals, tilting the scale towards enhanced cell death. Studies consistently demonstrate increased apoptotic activity in lung epithelial cells and alveolar macrophages from COPD patients, accompanied by an elevation of mitochondria-derived pro-apoptotic factors [30,31]. This dysregulation of apoptosis contributes significantly to tissue damage and perpetuates the chronic inflammatory state characteristic of COPD. Moreover, apoptotic cells release mitochondrial components into the extracellular milieu, acting as danger signals that exacerbate inflammation [32].

### 4.2. Mitophagy

Mitophagy, a crucial cellular recycling process, is intricately linked to mitochondrial health. Mitochondrial dysfunction can trigger dysfunctional mitophagy, leading to the accumulation of damaged mitochondria and cellular debris. In COPD, impaired mitophagy, a specific form of autophagy targeting mitochondria, is observed. This results in the persistence of damaged mitochondria, perpetuating oxidative stress and inflammation. The dysfunctional interplay between mitochondria and mitophagy contributes to the aberrant cellular processes seen in COPD, further compromising cellular homeostasis [33].

It is noteworthy that in the context of COPD, lung tissue damage can result in mitochondrial elongation, reflecting a failure to undergo normal mitophagy and remove injured mitochondria. The elongated mitochondria consist of a mix of both injured and healthy components, leading to reduced efficiency in ATP generation and the increased production of reactive oxygen species (ROS) [34,35,36]. Understanding mitochondria–endoplasmic reticulum dysfunctions and oxidative stress responses is essential for improving inflammatory and cellular dysfunctions exacerbated by viral infections, informing potential intervention strategies for these pathologic illnesses [34]. This additional perspective underscores the intricate relationship between mitophagy, mitochondrial dynamics, and the pathophysiology of COPD.

### 4.3. Cellular Senescence

Mitochondrial dysfunction is implicated in cellular senescence, a state of irreversible growth arrest. Senescent cells, which accumulate with age and in various pathologies, including COPD, contribute to tissue dysfunction [37,38]. Research indicates that mitochondrial dysfunction, particularly the release of mitochondrial DNA into the cytoplasm, activates cellular senescence pathways. Senescent cells, in turn, release pro-inflammatory signals and contribute to tissue remodeling. In the context of COPD, cellular senescence is observed in lung epithelial cells and fibroblasts, linking mitochondrial dysfunction to the senescence-associated secretory phenotype (SASP) and chronic inflammation [39]. Cigarette smoke-induced cellular senescence in chronic obstructive pulmonary disease (COPD) involves impaired mitophagy and the perinuclear accumulation of damaged mitochondria, highlighting a potential therapeutic target for chronic airway diseases [35] Chronic cigarette smoke (CS) exposure induces mitochondrial dysregulation, abnormal mitophagy, and lung cellular senescence, highlighting their roles in the pathogenesis of chronic obstructive pulmonary disease (COPD)/emphysema, as demonstrated using the mitoQC reporter mouse model [36].

### 4.4. Cross-Talk, Feedback Loops, and Translational Value

The intricate interplay of cellular events in mitochondrial dysfunction within COPD forms complex cross-talk and feedback loops. Dysregulated apoptosis contributes to cellular debris, presenting a challenge to the autophagy machinery. Simultaneously, senescent cells release inflammatory mediators that exacerbate mitochondrial dysfunction, establishing a self-perpetuating cycle [40,41]. This interconnected web of cellular consequences underscores the complexity of COPD pathology, emphasizing the necessity for holistic therapeutic approaches addressing various facets of mitochondrial dysfunction and its downstream effects. Understanding these intricate relationships serves as a foundation for developing targeted interventions capable of interrupting these feedback loops, potentially halting or slowing down the progression of COPD.

Recent clinical evidence from human subjects has significantly enhanced the translational relevance of mitochondrial dysregulation in COPD [4,42]. Studies utilizing advanced imaging techniques and molecular analyses in COPD patients have unequivocally confirmed the presence of mitochondrial abnormalities, establishing a direct link between genetic variations and disease manifestation. These findings underscore the clinical significance of comprehending mitochondrial genetics as a potential therapeutic target and emphasize the imperative need for further translational research to bridge the gap between laboratory discoveries and clinical applications [3,39,43,44,45].

## 5. Therapeutic Implications

Mitigating the impact of mitochondrial dysfunction in chronic obstructive pulmonary disease (COPD) holds significant promise for therapeutic interventions. This section explores potential strategies, including antioxidants, mitochondria-targeted compounds, and lifestyle modifications, aiming to restore mitochondrial health and ameliorate disease progression.

### 5.1. Therapeutic Implications of Mitochondrial Dysfunction in COPD

#### 5.1.1. Antioxidant Therapies

Oxidative stress, stemming from mitochondrial dysfunction, stands as a hallmark feature of COPD. Antioxidant therapies aim to counteract the excessive production of reactive oxygen species (ROS) and mitigate the downstream effects of oxidative damage. Common antioxidants, such as vitamins C and E, have been investigated for their potential to reduce oxidative stress in COPD patients. N-acetylcysteine (NAC), a precursor to the antioxidant glutathione, has demonstrated some efficacy in reducing exacerbations and improving lung function in COPD [46]. However, the overall impact of antioxidant therapies remains a subject of ongoing research, with challenges in achieving consistent and robust clinical benefits.

#### 5.1.2. Mitochondria-Targeted Compounds

Innovative strategies specifically targeting mitochondria have garnered attention as potential therapeutic interventions. Mitoquinone, a mitochondria-targeted antioxidant, has shown promise in preclinical studies by reducing oxidative stress and improving mitochondrial function. Similarly, Szeto–Schiller (SS) peptides, designed to enhance mitochondrial function, have demonstrated benefits in experimental models of lung disease [47]. Exploring compounds that selectively target mitochondrial dysfunction provides a tailored approach to mitigating the root causes of COPD. Clinical trials evaluating the safety and efficacy of these compounds are essential for establishing their therapeutic potential in a real-world setting.

#### 5.1.3. Lifestyle Modifications

Beyond pharmacological interventions, lifestyle modifications play a pivotal role in addressing mitochondrial dysfunction in COPD. Physical activity and exercise training have been shown to improve mitochondrial function and respiratory muscle strength, contributing to enhanced exercise tolerance and overall well-being in COPD patients. Dietary interventions, such as the adoption of a Mediterranean diet rich in antioxidants, omega-3 fatty acids, and polyphenols, may exert beneficial effects on mitochondrial health. Nutritional supplementation, particularly with coenzyme Q10, an essential component of the mitochondrial respiratory chain, has been explored for its potential to enhance mitochondrial function [48].

#### 5.1.4. Integrated Approaches

Combining therapeutic modalities targeting different aspects of mitochondrial dysfunction offers a holistic approach to COPD management. Integrated interventions may include a combination of antioxidant therapies, mitochondria-targeted compounds, and lifestyle modifications tailored to individual patient profiles.

Precision medicine approaches, guided by an understanding of the patient’s mitochondrial genetics and specific molecular abnormalities, could further enhance treatment efficacy. Personalized interventions may optimize therapeutic outcomes by addressing the unique characteristics of mitochondrial dysfunction in each individual. The exploration of integrated and personalized approaches represents a paradigm shift in COPD management, offering the potential for more effective and tailored strategies to improve patient outcomes and quality of life.

Integrating a discussion on COPD drugs, such as steroids and long-acting muscarinic antagonists (LAMA), and their impact on mitochondria could significantly enhance the clinical relevance of this work for healthcare professionals. Steroids, commonly used in COPD management to reduce inflammation and control exacerbations, have been shown to affect mitochondrial function [49]. Similarly, LAMA medications, which help relax the muscles around the airways to improve breathing, may also influence mitochondrial dynamics [50]. Understanding the mitochondrial implications of these drugs is crucial for optimizing treatment strategies and managing potential side effects in COPD patients. By exploring the interplay between medication regimens and mitochondrial health, this research can provide valuable insights that bridge the gap between basic science and clinical practice, ultimately improving patient outcomes.

### 5.2. Challenges and Future Directions

Despite the promising avenues for therapeutic interventions, challenges in translating preclinical findings into clinically effective treatments persist. Heterogeneity in COPD presentations, patient responses, and the multifaceted nature of mitochondrial dysfunction necessitate a nuanced approach to therapeutic development. Long-term studies evaluating the sustained efficacy and safety of interventions are critical for establishing their role in COPD management. Moreover, identifying biomarkers that reflect changes in mitochondrial function and guide treatment decisions remains an ongoing research priority. Future directions should explore emerging technologies, such as mitochondrial transplantation and gene therapies, to directly address mitochondrial dysfunction at the cellular level [41]. Additionally, collaborative efforts between researchers, clinicians, and pharmaceutical industries are essential for advancing novel therapeutics from bench to bedside.

## 6. Future Directions in Understanding and Addressing Mitochondrial Dysfunction in COPD

As we stand on the precipice of advancing our understanding of mitochondrial dysfunction in chronic obstructive pulmonary disease (COPD), several future directions beckon, offering new avenues for research, diagnosis, and treatment.

### 6.1. Identification of Novel Mitochondrial Biomarkers

The discovery and validation of specific mitochondrial biomarkers hold the potential to revolutionize COPD diagnostics and prognostics. Biomarkers associated with mitochondrial dysfunction could serve as early indicators of disease onset, guide personalized treatment decisions, and monitor treatment responses. Continued efforts to identify and validate such biomarkers will refine our ability to assess and manage COPD at the molecular level [51].

### 6.2. Advancements in Mitochondria-Targeted Therapies

The evolution of mitochondria-targeted therapies remains a dynamic area of research. Investigating novel compounds, peptides, and nanoparticles with enhanced selectivity for mitochondria could yield more potent and specific interventions. Preclinical studies and early-phase clinical trials exploring the safety and efficacy of these innovative therapies are crucial for translating laboratory findings into tangible benefits for COPD patients [43].

### 6.3. Integration of Omics Technologies

Integrating multi-omics technologies, encompassing genomics, transcriptomics, proteomics, and metabolomics, will provide a comprehensive view of the molecular landscape of COPD [52]. Applying these high-throughput approaches to large cohorts of COPD patients will uncover intricate molecular signatures, allowing for the identification of subtypes and the development of personalized therapeutic strategies based on the unique molecular characteristics of each individual.

### 6.4. Patient-Centric Approaches

Future research should prioritize patient-centric approaches that consider the diverse phenotypes and endotypes within the COPD spectrum [53]. Integrating patient-reported outcomes, quality of life assessments, and real-world data into research frameworks will ensure that interventions are not only scientifically rigorous but also meaningful and impactful for individuals living with COPD.

### 6.5. Technological Innovations and Collaborative Efforts

Technological advancements, such as the integration of artificial intelligence and machine learning, can enhance our ability to analyze vast datasets, identify patterns, and predict disease trajectories [54]. Collaborative efforts between academia, industry, and healthcare providers will be instrumental in translating research findings into tangible improvements in COPD care, fostering a continuum of innovation and discovery. These collaborative endeavors will facilitate the development of novel diagnostic tools, therapeutic interventions, and holistic management strategies that address the multifaceted nature of COPD, ultimately improving patient outcomes and quality of life.

## 7. Conclusions: Unraveling the Mitochondrial Tapestry in COPD

The exploration of mitochondrial dysfunction in chronic obstructive pulmonary disease (COPD) has illuminated a complex and interconnected tapestry of molecular events that significantly contribute to disease pathogenesis. From the intricate dance of oxidative stress and bioenergetic impairment to the profound consequences on apoptosis, autophagy, and cellular senescence, mitochondria emerge as central orchestrators in the COPD narrative (Table 1). As we navigate through this landscape, the therapeutic implications provide a glimmer of hope for transforming COPD management (Table 2). Antioxidant therapies, mitochondria-targeted compounds, and lifestyle modifications offer promising avenues to mitigate the impact of mitochondrial dysfunction, providing a beacon for improved patient outcomes. The integration of these approaches, tailored to individualized needs and guided by an understanding of mitochondrial genetics, holds the potential to revolutionize COPD care. Looking ahead, future directions beckon us to delve deeper into the molecular intricacies of COPD, identifying novel biomarkers, advancing mitochondria-targeted therapies, and embracing patient-centric approaches. The integration of omics technologies and collaborative efforts promises a nuanced understanding of COPD’s heterogeneity, paving the way for personalized interventions that resonate with the unique molecular signatures of each patient. In conclusion, this journey through the mitochondrial dimensions of COPD culminates in a call for continued research, innovation, and collaboration. The relentless pursuit of knowledge in this field offers a beacon of hope for the millions affected by COPD, envisioning a future where precision medicine, advanced therapeutics, and patient-centric care converge to redefine the landscape of COPD management. As we unravel the mitochondrial tapestry in COPD, it provides not merely a conclusion but a prelude to a new chapter—one that holds the promise of breakthroughs, advancements, and a profound impact on the lives of those navigating the challenges of COPD.

## Figures and Tables

**Table 1:** Summary of mitochondrial dysfunction in COPD.

	Mechanisms	Consequences
Oxidative Stress	Mitochondrial dysfunction leads to increased production of reactive oxygen species (ROS), creating an imbalance in the cellular redox state. This imbalance perpetuates inflammation and oxidative damage, contributing to the pathophysiology of COPD.	The consequences of oxidative stress include damage to cellular components and structures, further exacerbating inflammation and initiating a cascade of events that contribute to COPD progression.
Bioenergetic Impairment	Alterations in mitochondrial respiratory chain function, including decreased electron transport chain complex activity, lead to insufficient ATP production, impacting cellular processes critical for maintaining lung health and function.	Bioenergetic deficits affect mucociliary clearance, immune responses, and tissue repair, contributing to the systemic manifestations of COPD, such as skeletal muscle dysfunction and exercise intolerance.
Mitochondrial Genetics	Specific mutations in mtDNA are associated with increased risk of COPD development. These mutations contribute to impaired mitochondrial function, leading to oxidative stress and energy imbalance, key features of COPD pathology.	Certain mitochondrial haplogroups are linked to COPD susceptibility and severity. Understanding these haplogroups provides insights into the genetic influences beyond individual mtDNA mutations.
Cellular Consequences	Mitochondrial dysfunction in COPD disrupts the balance of apoptosis, leading to increased apoptotic activity in lung epithelial cells and alveolar macrophages. Impaired mitophagy, a specific form of autophagy targeting mitochondria, is observed in COPD. Mitochondrial dysfunction activates cellular senescence pathways, leading to irreversible growth arrest.	Apoptosis contributes to tissue damage and perpetuates the chronic inflammatory state characteristic of COPD. Mitophagy results in the persistence of damaged mitochondria, perpetuating oxidative stress and inflammation. Senescent cells release pro-inflammatory signals, contributing to the tissue remodeling and chronic inflammation observed in COPD.

**Table 2:** Therapeutic implications and future directions of mitochondrial dysfunction in COPD.

	Therapeutic Implications	Future Directions
Oxidative Stress	Recognizing the pivotal role of oxidative stress suggests potential therapeutic strategies targeting antioxidant pathways to mitigate the detrimental effects of oxidative damage in COPD. Physical activity, exercise training, and dietary interventions, such as adopting a diet rich in antioxidants, omega-3 fatty acids, and polyphenols, are explored for their potential to improve mitochondrial health and reduce oxidative stress.	Exploring innovative compounds specifically targeting mitochondria to address the root causes of oxidative stress. Mitoquinone, a mitochondria-targeted antioxidant, and Szeto–Schiller (SS) peptides are among the compounds showing promise in preclinical studies.
Bioenergetic Impairment	Addressing bioenergetic impairment in COPD involves strategies to restore mitochondrial function and enhance cellular energy production. Potential interventions include promoting mitochondrial biogenesis, improving ATP synthesis pathways, and supporting overall mitochondrial health. Physical exercise, pharmacological agents, and nutritional interventions may play a role in stimulating mitochondrial biogenesis.	Elucidating the specific mechanisms underlying bioenergetic impairment in COPD, identifying novel targets for intervention, and conducting robust clinical trials to evaluate the safety and efficacy of emerging therapies targeting mitochondrial function.
Mitochondrial Genetics	Navigating the genetic landscape, and elucidating the role of mitochondrial DNA mutations, variations, and haplogroups in COPD susceptibility and severity. Therapies specifically targeting mitochondrial DNA mutations or variations could emerge as disease-modifying agents in COPD. Gene-editing technologies, such as CRISPR-Cas9, may hold potential for correcting mitochondrial genetic defects. Emerging technologies like mitochondrial replacement therapies (MRT) involve replacing defective mitochondria with healthy ones. Advancements in gene and cell therapies may offer targeted interventions for mitochondrial genetic abnormalities.	Expanding our understanding of the functional consequences of mitochondrial genetic variations in COPD, exploring novel therapeutic targets, and advancing technologies for the precise manipulation of mitochondrial DNA.
Cellular Consequences	Examining cellular consequences, including apoptosis, autophagy, and cellular senescence, providing insights into mechanisms linking mitochondrial dysfunction to COPD pathology. Addressing the cross-talk between apoptosis, autophagy, and senescence is crucial. Therapies that target multiple pathways simultaneously may disrupt the self-perpetuating cycle of cellular consequences observed in COPD. Integrated therapeutic approaches may involve a combination of anti-apoptotic, pro-autophagic, and senolytic agents, tailored to individual patient profiles. A multi-modal approach targeting different cellular consequences simultaneously may enhance therapeutic efficacy.	Future research should explore the intricate molecular mechanisms of cellular consequences in COPD in preclinical and clinical settings, identifying novel therapeutic targets and assessing the safety and efficacy of emerging interventions.

## Data Availability

No new data were created in this study. Data sharing is not applicable to this article.

## Abbreviations

COPD: chronic obstructive pulmonary disease.

## References

[1] Agustí A., Celli B.R., Criner G.J., Halpin D., Anzueto A., Barnes P., Bourbeau J., Han M.K., Martinez F.J., Montes de Oca M. (2023). Global initiative for chronic obstructive lung disease 2023 report: GOLD executive summary. Am. J. Respir. Crit. Care Med..

[2] Agustí A., Melen E., DeMeo D.L., Breyer-Kohansal R., Faner R. (2022). Pathogenesis of chronic obstructive pulmonary disease: Understanding the contributions of gene—Environment interactions across the lifespan. Lancet Respir. Med..

[3] Dailah H.G. (2022). Therapeutic potential of small molecules targeting oxidative stress in the treatment of chronic obstructive pulmonary disease (COPD): A comprehensive review. Molecules.

[4] Wiegman C.H., Michaeloudes C., Haji G., Narang P., Clarke C.J., Russell K.E., Bao W., Pavlidis S., Barnes P.J., Kanerva J. (2015). Oxidative stress–induced mitochondrial dysfunction drives inflammation and airway smooth muscle remodeling in patients with chronic obstructive pulmonary disease. J. Allergy Clin. Immunol..

[5] Di Meo S., Reed T.T., Venditti P., Victor V.M. (2016). Harmful and beneficial role of ROS. Oxid. Med. Cell. Longev..

[6] Antunes M.A., Lopes-Pacheco M., Rocco P.R. (2021). Oxidative stress-derived mitochondrial dysfunction in chronic obstructive pulmonary disease: A concise review. Oxidative Med. Cell. Longev..

[7] Maremanda K.P., Sundar I.K., Rahman I. (2021). Role of inner mitochondrial protein OPA1 in mitochondrial dysfunction by tobacco smoking and in the pathogenesis of COPD. Redox Biol..

[8] Boukhenouna S., Wilson M.A., Bahmed K., Kosmider B. (2018). Reactive oxygen species in chronic obstructive pulmonary disease. Oxidative Med. Cell. Longev..

[9] Rahman I., Adcock I. (2006). Oxidative stress and redox regulation of lung inflammation in COPD. Eur. Respir. J..

[10] Neofytou E., Tzortzaki E.G., Chatziantoniou A., Siafakas N.M. (2012). DNA damage due to oxidative stress in chronic obstructive pulmonary disease (COPD). Int. J. Mol. Sci..

[11] Guo C., Sun L., Chen X., Zhang D. (2013). Oxidative stress, mitochondrial damage and neurodegenerative diseases. Neural Regen. Res..

[12] Kosmider B., Lin C.-R., Karim L., Tomar D., Vlasenko L., Marchetti N., Bolla S., Madesh M., Criner G.J., Bahmed K. (2019). Mitochondrial dysfunction in human primary alveolar type II cells in emphysema. eBioMedicine.

[13] Barnes P.J. (2020). Oxidative stress-based therapeutics in COPD. Redox Biol..

[14] Forman H.J., Zhang H. (2021). Targeting oxidative stress in disease: Promise and limitations of antioxidant therapy. Nat. Rev. Drug Discov..

[15] Ryan E.M., Sadiku P., Coelho P., Watts E.R., Zhang A., Howden A.J., Sanchez-Garcia M.A., Bewley M., Cole J., McHugh B.J. (2023). NRF2 Activation Reprograms Defects in Oxidative Metabolism to Restore Macrophage Function in Chronic Obstructive Pulmonary Disease. Am. J. Respir. Crit. Care Med..

[16] Cloonan S.M., Choi A.M. (2016). Mitochondria in lung disease. J. Clin. Investig..

[17] Wang Y., Li P., Cao Y., Liu C., Wang J., Wu W. (2023). Skeletal Muscle Mitochondrial Dysfunction in Chronic Obstructive Pulmonary Disease: Underlying Mechanisms and Physical Therapy Perspectives. Aging Dis..

[18] Haji G., Wiegman C.H., Michaeloudes C., Patel M.S., Curtis K., Bhavsar P., Polkey M.I., Adcock I.M., Chung K.F., COPDMAP consortium (2020). Mitochondrial dysfunction in airways and quadriceps muscle of patients with chronic obstructive pulmonary disease. Respir. Res..

[19] Seymour J., Spruit M., Hopkinson N., Natanek S., Man W.-C., Jackson A., Gosker H., Schols A., Moxham J., Polkey M. (2010). The prevalence of quadriceps weakness in COPD and the relationship with disease severity. Eur. Respir. J..

[20] Pokharel M.D., Garcia-Flores A., Marciano D., Franco M.C., Fineman J.R., Aggarwal S., Wang T., Black S.M. (2024). Mitochondrial network dynamics in pulmonary disease: Bridging the gap between inflammation, oxidative stress, and bioenergetics. Redox Biol..

[21] Nam H.-S., Izumchenko E., Dasgupta S., Hoque M.O. (2017). Mitochondria in chronic obstructive pulmonary disease and lung cancer: Where are we now?. Biomark. Med..

[22] MacNee W. (2016). Is chronic obstructive pulmonary disease an accelerated aging disease?. Ann. Am. Thorac. Soc..

[23] Liu G., Summer R. (2019). Cellular metabolism in lung health and disease. Annu. Rev. Physiol..

[24] Zheng S., Wang C., Qian G., Wu G., Guo R., Li Q., Chen Y., Li J., Li H., He B. (2012). Role of mtDNA haplogroups in COPD susceptibility in a southwestern Han Chinese population. Free Radic. Biol. Med..

[25] Liu J., Wang J., Liu Y., Li G., He X. (2023). Mitochondrial quality control in lung diseases: Current research and future directions. Front. Physiol..

[26] Liu S.-F., Kuo H.-C., Tseng C.-W., Huang H.-T., Chen Y.-C., Tseng C.-C., Lin M.-C. (2015). Leukocyte mitochondrial DNA copy number is associated with chronic obstructive pulmonary disease. PLoS ONE.

[27] Liu S.-F., Chang H.-C., Chang Y.-P., Kuo H.-C., Tsai Y.-C. (2022). IL13 Promoter (−1055) Polymorphism Associated with Leukocyte Mitochondria DNA Copy Number in Chronic Obstructive Pulmonary Disease. Cells.

[28] Jiang Z.-M., Zhao Q.-S., Xu Y.-Q., Li T., Jie J., Zhang Z.-H. (2016). Whole mitochondrial genome sequencing and analysis for chronic obstructive pulmonary disease rat strain. Mitochondrial DNA Part A.

[29] Lin Q., Zhang C.-F., Chen J.-Y., Guo Z.-K., Wu S.-Y., Li H.-Y. (2023). Targeting Mitochondrial Dysfunction With LncRNAs in a Wistar Rat Model of Chronic Obstructive Pulmonary Disease. Vivo.

[30] Plataki M., Tzortzaki E., Rytila P., Demosthenes M., Koutsopoulos A., Siafakas N.M. (2006). Apoptotic mechanisms in the pathogenesis of COPD. Int. J. Chronic Obstr. Pulm. Dis..

[31] Demedts I.K., Demoor T., Bracke K.R., Joos G.F., Brusselle G.G. (2006). Role of apoptosis in the pathogenesis of COPD and pulmonary emphysema. Respir. Res..

[32] Hikichi M., Mizumura K., Maruoka S., Gon Y. (2019). Pathogenesis of chronic obstructive pulmonary disease (COPD) induced by cigarette smoke. J. Thorac. Dis..

[33] Tsubouchi K., Araya J., Kuwano K. (2018). PINK1-PARK2-mediated mitophagy in COPD and IPF pathogeneses. Inflamm. Regen..

[34] Manevski M., Muthumalage T., Devadoss D., Sundar I.K., Wang Q., Singh K.P., Unwalla H.J., Chand H.S., Rahman I. (2020). Cellular stress responses and dysfunctional Mitochondrial–cellular senescence, and therapeutics in chronic respiratory diseases. Redox Biol..

[35] Ahmad T., Sundar I.K., Lerner C.A., Gerloff J., Tormos A.M., Yao H., Rahman I. (2015). Impaired mitophagy leads to cigarette smoke stress-induced cellular senescence: Implications for chronic obstructive pulmonary disease. FASEB J..

[36] Wang Q., Unwalla H., Rahman I. (2022). Dysregulation of mitochondrial complexes and dynamics by chronic cigarette smoke exposure utilizing MitoQC reporter mice. Mitochondrion.

[37] Vasileiou P.V., Evangelou K., Vlasis K., Panayiotidis M.I., Chronopoulos E., Passias P.-G., Kouloukoussa M., Gorgoulis V.G., Havaki S. (2019). Mitochondrial homeostasis and cellular senescence. Cells.

[38] Korolchuk V.I., Miwa S., Carroll B., Von Zglinicki T. (2017). Mitochondria in cell senescence: Is mitophagy the weakest link?. EBioMedicine.

[39] Birch J., Barnes P.J., Passos J.F. (2018). Mitochondria, telomeres and cell senescence: Implications for lung ageing and disease. Pharmacol. Ther..

[40] Lerner C.A., Sundar I.K., Rahman I. (2016). Mitochondrial redox system, dynamics, and dysfunction in lung inflammaging and COPD. Int. J. Biochem. Cell Biol..

[41] Wang J., Li H., Yao Y., Zhao T., Chen Y.-Y., Shen Y.-L., Wang L.-L., Zhu Y. (2018). Stem cell-derived mitochondria transplantation: A novel strategy and the challenges for the treatment of tissue injury. Stem Cell Res. Ther..

[42] Mumby S., Adcock I.M. (2022). Recent evidence from omic analysis for redox signalling and mitochondrial oxidative stress in COPD. J. Inflamm..

[43] Fairley L.H., Das S., Dharwal V., Amorim N., Hegarty K.J., Wadhwa R., Mounika G., Hansbro P.M. (2023). Mitochondria-Targeted Antioxidants as a Therapeutic Strategy for Chronic Obstructive Pulmonary Disease. Antioxidants.

[44] Chellappan D.K., Paudel K.R., Tan N.W., Cheong K.S., Khoo S.S.Q., Seow S.M., Chellian J., Candasamy M., Patel V.K., Arora P. (2022). Targeting the mitochondria in chronic respiratory diseases. Mitochondrion.

[45] Zhou W.-C., Qu J., Xie S.-Y., Sun Y., Yao H.-W. (2021). Mitochondrial dysfunction in chronic respiratory diseases: Implications for the pathogenesis and potential therapeutics. Oxidative Med. Cell. Longev..

[46] Dekhuijzen P., Van Beurden W. (2006). The role for N-acetylcysteine in the management of COPD. Int. J. Chronic Obstr. Pulm. Dis..

[47] Sheu S.-S., Nauduri D., Anders M. (2006). Targeting antioxidants to mitochondria: A new therapeutic direction. Biochim. Biophys. Acta (BBA)-Mol. Basis Dis..

[48] Chan S.M., Selemidis S., Bozinovski S., Vlahos R. (2019). Pathobiological mechanisms underlying metabolic syndrome (MetS) in chronic obstructive pulmonary disease (COPD): Clinical significance and therapeutic strategies. Pharmacol. Ther..

[49] Kokkinopoulou I., Moutsatsou P. (2021). Mitochondrial glucocorticoid receptors and their actions. Int. J. Mol. Sci..

[50] De Sarno P., Shestopal S.A., King T.D., Zmijewska A., Song L., Jope R.S. (2003). Muscarinic receptor activation protects cells from apoptotic effects of DNA damage, oxidative stress, and mitochondrial inhibition. J. Biol. Chem..

[51] Li L., Qi R., Zhang L., Yu Y., Hou J., Gu Y., Song D., Wang X. (2021). Potential biomarkers and targets of mitochondrial dynamics. Clin. Transl. Med..

[52] Blutt S.E., Coarfa C., Neu J., Pammi M. (2023). Multiomic Investigations into Lung Health and Disease. Microorganisms.

[53] Gea J., Enríquez-Rodríguez C.J., Pascual-Guardia S. (2023). Metabolomics in COPD. Arch. Bronconeumol..

[54] Feng Y., Wang Y., Zeng C., Mao H. (2021). Artificial intelligence and machine learning in chronic airway diseases: Focus on asthma and chronic obstructive pulmonary disease. Int. J. Med. Sci..

